# Identification of Barriers Preventing Biosimiliar Oncology Medication Adoption

**DOI:** 10.3390/medicina58111533

**Published:** 2022-10-27

**Authors:** John Hair, Thomas Maryon, Cristian Lieneck

**Affiliations:** 1 Health Administration Division, School of Health Sciences, The Herbert H. & Grace A. Dow College of Health Professions, Central Michigan University, Mount Pleasant, MI 48859, USA; 2 School of Health Administration, Texas State University, San Marcos, TX 78666, USA

**Keywords:** oncology, cancer, biosimilar, barriers, access, obstacles

## Abstract

(1) *Background:* A biosimilar is a biologic medical product that has been approved by the United States Food and Federal Drug Administration (FDA) and is an almost identical copy of an original biologic product yet manufactured by a different company. Biosimilars are often assumed to be the same as generic medications, while often made from living organisms. Through clinical trials, biosimilars have been shown to be both as safe and as effective as their originator products. Biosimilars have also proven they can reduce the costs to both insurance companies and patients in many circumstances. However, despite their cost savings, biosimilar manufacturers continue to face barriers in having oncologists and cancer centers prescribe them for their patients. This review aims to identify barriers associated with medical provider prescriptive behaviors related to biosimilars for patients. (2) *Methods:* Reviewers analyzed 27 articles and identified common themes. (3) *Results:* After a thorough literature review, the researchers identified seven barriers to prescribing of biosimilars: physician comfort in originators instead of biosimilars, patient reluctance to switch from a current biologic to a biosimilar, provider profits associated with an originator biologic, lack of stakeholder education on biosimilars, lack of provider team knowledge of biosimilars, lack of knowledge surrounding the biosimilar FDA approval process, and hesitancy to stock multiple drugs for a specific indication. (4) *Conclusions:* This review’s findings of identified barriers to use of biosimilars provides insight for healthcare providers and organizations surrounding prescribing practices and potential treatment benefits for cancer patients who may benefit from biosimilar treatment medications.

## 1. Introduction

### 1.1. Background and Rationale

Biosimilars are FDA-approved medications that are biological in type and contain a very similar substance that has been previously approved by the FDA. Similar to other generic medications, biosimilars offer an opportunity for lower healthcare costs due to intrinsic price competition with the originator (reference) product. Providers and healthcare organizations require an understanding for biosimilars and their appropriate use in order to know how to implement these medications into their practice and how to explain these medications to patients. In this vein, an understanding of how biosimilars are regulated by the FDA, approved, and even paid for is important for all stakeholders involved in the treatment of cancer and associated diseases. The federal government and the insurance companies have heavily pressed for the use of biosimilars in the healthcare arena.

Dating back to 1984 when the Drug Price Competition and Patient Term Restoration Act (Hatch–Waxman Amendments) became a law, the use of FDA-approved generic medications was permitted without having to repeat the original research previously established by the brand drug’s safety and efficacy results. At the time of approval, many medications were chemically synthesized; therefore, generic medications based upon the originator medication were structurally identical. However, ongoing innovation in the pharmaceutical industry would challenge this brand-generic structural component similarity, with Eli Lilly developing a method to use bacteria to synthesize insulin utilizing recombinant DNA. As a result of this research effort, the biologics drug category was established, differentiating from other medications by involving the use of living organisms [1].

Decades later, the biosimilar drug class continues to successfully reproduce their originator medication’s components and efficacy, serving as a biologic generic medication alternative. These biosimilars contain similar versions of the active living substance and components of their originator biologic medication (originator product), while offering an FDA-approved quality, safety, immunogenicity, and treatment efficacy in comparative studies [1].

The financial impact of biosimilar competition can be dramatic, especially within the oncology realm of medications. However, oncologists’ understanding of biosimilars is critical to them prescribing more biosimilars moving forward. Both the oncologist and the cancer center need to comprehend how biosimilars are created, the safety and efficacy of the products, and the clinical trials they must undergo prior to approval. Education of the nurses and staff, as well as the patients, regarding biosimilars is equally important. Before oncologists are willing to prescribe biosimilars on a consistent basis all shareholders must be willing to work together to identify the barriers to prescribing them and then create solutions to overcome the barriers. 

### 1.2. Objectives

The objective of this review was to determine the barriers that are preventing biosimilars from being adopted/prescribed by oncologists and cancer centers. An additional goal was to understand what factors are influencing physicians and cancer centers to continue to prescribe the originator product when biosimilars are available at a significantly reduced cost. It is imperative to understand where the educational gaps are when explaining the biosimilar benefits to healthcare providers, as well as oncology patients. By focusing on these barriers in the literature, the hope is to provide a clear understanding of the ways to overcome the barriers in these situations. The goal is also to offer suggestions that physicians and cancer centers can implement to help increase the amount of biosimilars that they are prescribing and to reduce costs for managed care companies, as well as to the patients themselves. The move forward from originator products to biosimilars could lead to significant savings to the healthcare industry. In Europe, 37 biosimilars oncology medications have been approved, with a mean price discount of 15–40% as compared to their originators [1].

## 2. Materials and Methods

### 2.1. Eligibility Criteria

Studies were included in this review if they focused on obstacles and barriers experienced by physicians and cancer centers when it comes to prescribing biosimilars. The articles had to be published in quality peer-reviewed journals. Articles that included barriers that were not experienced at the physician/prescriber level were not included in the review. Articles needed to be written in English and had to be a United States provider-based study to be included. Articles were required to be published between 1 January 2011 and 1 June 2022. Any articles that focused on only one specific oncology medication were excluded and were deemed to be not broad enough for the purpose of this review.

### 2.2. Information Sources and Search Details

Two databases were queried to identify the review articles: Cumulative Index to Nursing and Allied Health Literature (CINAHL) and PubMed (which queries MEDLINE) The database search was conducted from 15 May through 15 June 2022. The goal of the search was to identify United States provider-based studies, written in English, and including medications falling within the entire class of possible biosimilar oncology prescriptions. While researchers acknowledge the importance of a global perspective of biosimilars, the goal of this paper is to specifically consider the United States healthcare system and the barriers that providers face in the United States. The goal was not to use studies that looked specifically at one or two medications and their biosimilars. The final search term thread used was [“oncology biosimilar barriers” OR “oncology biosimilar access” OR “oncology biosimilar obstacles” OR “cancer biosimilar barriers” OR “cancer biosimilar obstacles”]. This search string provided the maximum number of relevant search results identified by the research team after multiple research database queries.

### 2.3. Initial Study Selection

The review was guided by the Preferred Reporting Items for Systematic Reviews and Meta-Analysis (PRISMA). The initial database search included any/all identified articles to be included in the study, regardless of whether a full-text version of any article was available. The “full text only” search criterion was purposely not selected in the initial database search, which allowed for a maximum number of initial articles to be identified. Use of the home institution’s (Central Michigan University) research database access privileges permitted access to the full text of all the identified articles for the collective process. An Excel spreadsheet was utilized to categorize and rate each article regarding inclusion criteria. On the basis of the abstract screening and full article review, researchers were able to decide which articles met the criteria for inclusion into the systematic review.

## 3. Results

### 3.1. Study Selection and Exclusion Process

**Figure 1:**
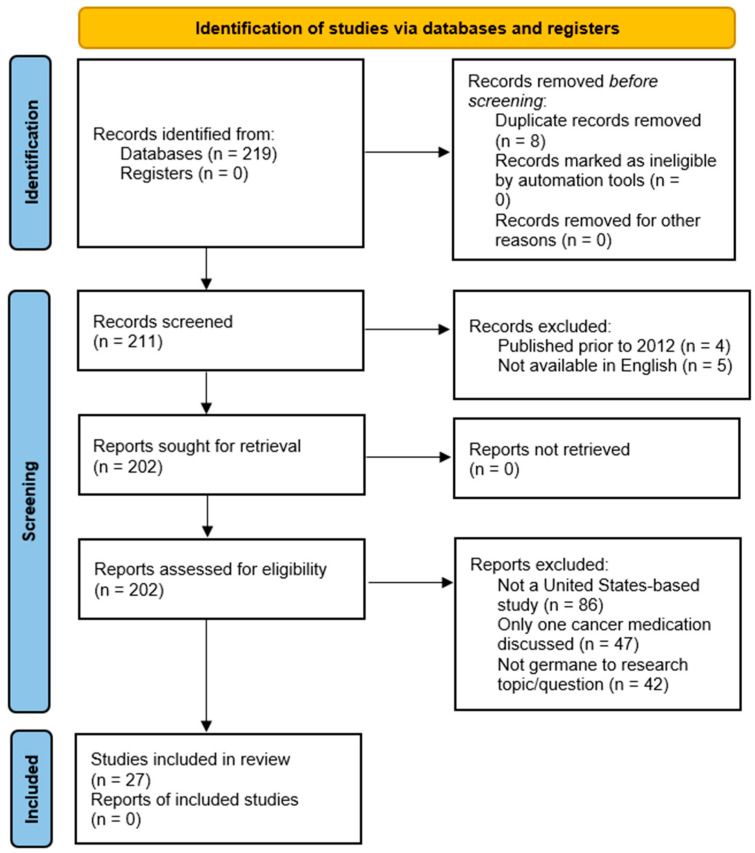
Preferred Reporting Items for Systematic Reviews and Meta-Analyses (PRISMA) flowchart demonstrating the study selection process.

Figure 1 demonstrates the study selection and the exclusion process, which initially identified 219 articles from the two research databases. Eight duplicates were identified and removed from the search. Of the 211 articles left, four were removed because they were published before 2011, and five were removed because they were not published in English. That left a total of 175 articles removed for the following reasons:

(a) Not a United States provider-based study (86 articles),
(b) Only discussed one specific cancer medication (47 articles),
(c) Not related to the research question (42 articles).

This action did not result in any additional articles being included in the review beyond those articles previously identified. Upon completion of the review, a total of 27 articles were included in the review.

### 3.2. Study Characteristics

Reviews entailed a systematic approach to identifying barriers associated with oncologists and cancer centers prescribing biosimilars medications to their patients when one is available. Barriers found during the systematic review are summarized in Table 1.

**Figure 2:**
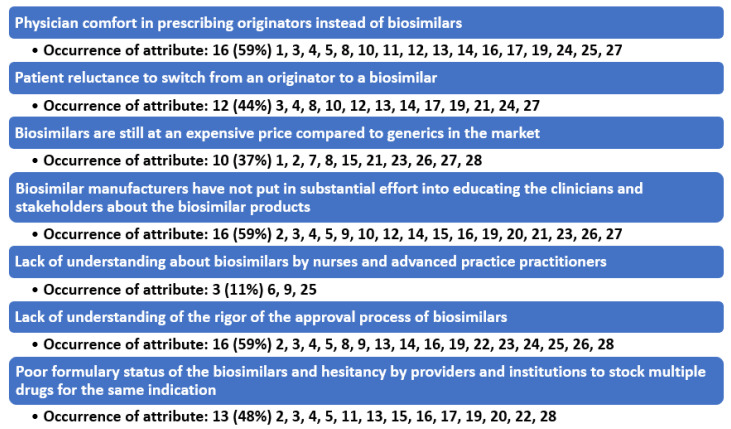
Identified themes (constructs) identified as barriers to the implementation of biosimilar oncology medications.

Results of the review process demonstrated seven barrier themes in the literature that are preventing the adoption of biosimilars into the prescribing habits of oncologists (Figure 2).

## 4. Discussion

### 4.1. Summary of Evidence

The FDA established additional guidance in 2017 surrounding biosimilars, now requiring the drug manufacturers to conduct a switching study (or studies) in order to ensure that replacement of the originator medication with the biosimilar remains safe and effective [3]. Furthermore, the biosimilar has to also demonstrate the same clinical outcomes as the originator reference medication. Once established and approved, such interchangeability of the originator drug with the biosimilar was able to be promoted and offered as approved status for biosimilar substitution [3]. Even with these changes, oncologists and cancer centers continue to face numerous barriers when they go to prescribe biosimilars.

Seven primary themes (constructs) were found to be associated with the barriers that are preventing biosimilars from being prescribed for more patients. One identified barrier to prescribing biosimilars and percentage of attribute occurrences is physician comfort in prescribing reference biologics instead of biosimilars. A second barrier is patient reluctance to switch from a biologic to a biosimilar. A third is the fact that biosimilars, while cheaper than the originator, are still at a high price due to an overall lack of competition. Therefore, physicians and institutions can have more profitability by using the originator biologic. A fourth major obstacle is biosimilar manufacturers’ lack of substantial effort in educating stakeholders and allowing the originator biologics to counter detail their products. A fifth barrier is lack of understanding about biosimilars by nurses and advanced practice practitioners. Barrier number six is lack of understanding of the rigor of the approval process of biosimilars and patients and providers perceiving lack of parity in biosimilars and support services. A seventh barrier is formulary status of the biosimilars and hesitancy by providers and institutions to stock multiple drugs for the same indication.

### 4.2. Barrier to Prescribing #1: Physician Comfort in Prescribing Originators Instead of Biosimilars

Many physicians have concerns about efficacy of biosimilars and whether they have been through significant clinical trials for their patients diagnosed with cancer. Surveys of oncologists emphasized that rigorous regulatory standards are needed for clinicians to consider biosimilars as acceptable alternatives to the standard of care. A 2018 statement by the American Society of Clinical Oncology (ASCO) commented on the use of biosimilars in clinical practice. This paper identified the need for post marketing evidence development to enhance physician and patient confidence in their use [4]. A group of 327 specialists were asked why they do not prescribe biosimilars more often, and the most common situations that they would not prescribe a biosimilar were where there was a lack of clinical data supporting efficacy (32%) or evidence of adverse effects (17%) [10]. Biosimilars are often viewed as being “generics” by physicians and patients that do not understand the rigorous clinical trials that biosimilars must undergo before they receive FDA approval. Biosimilars will be routinely prescribed only when clinicians are convinced of their safety and efficacy.

### 4.3. Barrier to Prescribing #2: Patient Reluctance to Switch from a Biologic to a Biosimilar

Patients want to be shown data demonstrating that the clinical trials were efficacious and safe. Biosimilar companies have failed to create marketing material for patients so that they can find information about biosimilars. Out-of-pocket expenses for the patients have continued to be a barrier. In the USA, patient out-of-pocket costs for intravenous cancer drugs have increased substantially in recent years. This continues to restrict the ability of patients to even access biosimilars because they are still considered intravenous cancer drugs. Biosimilar companies have failed to create financial assistance programs to help patients that have substantial insurance deductibles or simply have no insurance at all. Another limiting factor to biosimilars is a modest number of drug manufacturers that have the complex research and development capabilities to advance a biosimilar to market. This means that there has not been an exceptional amount of competition in the oncology biosimilar market. Therefore, it is unlikely that the competition dynamics for biosimilars will echo those of the small-molecule drug market.

### 4.4. Barrier to Prescribing #3: Biosimilars Are Still at a High Price and Physicians and Institutions Can Have More Profitability by Using the Originator Biologic

One of the barriers that physicians face with biosimilars is the complex and dynamic CMS reimbursement rules for biosimilars which create confusion for billing offices. Physicians often tend to prescribe originator products because they are familiar with the reimbursement rules and do not want to risk losing on reimbursement by prescribing products that they are not familiar with. The reimbursement of a drug is tied to the average sales price (ASP). As a result, a higher ASP leads to a higher reimbursement (Medicare Part B is set at 104.3% of ASP). In this system, a biosimilar with a lower ASP delivers a lower reimbursement than its reference product with a higher ASP. Most cancer centers are “buy and bill” facilities, which indicates the facility buys the cancer medications and bills for them after they administer them to the patients. This system allows the center to receive higher profits for medications that charge more, which is generally the originator product.

### 4.5. Barrier to Prescribing #4: Biosimilar Manufacturers Have Not Put in Substantial Effort into Educating Stakeholders and Many of the Originator Biologic Companies Are Counter Detailing the Biosimilar Products

Medical providers and patients alike both express concerns with “switching” between the originator biologic and a biosimilar alternative. Furthermore, a changing back and forth between the originator product and the biosimilar agent has also been identified as a provider and patient concern, especially when the reason for the switch is cost-oriented (known as “nonmedical switching”). A 2019 survey sponsored by a reference product manufacturer found that 84% of US physicians were opposed to switching a stable patient [26].

Physicians are willing to place patients on biosimilars when there is proven efficacy and cost savings; however, they are extremely hesitant to switch patients when they do not have the clinical trial data readily available. Nearly all originator products have marketing representatives in the physician offices consulting with the physicians and sharing the newest clinical trials. Rarely have the biosimilar companies paid the money to have representatives in the clinical offices sharing the newest clinical data about biosimilar products with the oncologists. As a result, the acceptance of biosimilars is significantly related to a lower perceived time to explain a biosimilar to a patient and lower number of weekly patient appointments. Oncologists lack the literature from the biosimilar manufacturers to hand over to their patients to tell them about the biosimilar and the benefits.

Physicians perceive that they lack time with their patients and without resources to explain why they are switching to biosimilars, and they are extremely hesitant to prescribe the biosimilar. The impact of “nocebo” effects, whereby negative expectations of treatment lead to potentially worse outcomes, also needs to be considered by healthcare providers. These effects are frequently caused because patients simply perceive that a drug is less expensive because it is less effective [14].

### 4.6. Barrier to Prescribing #5: Lack of Understanding about Biosimilars by Nurses and Advanced Practice Practitioners

Advanced practitioner providers play a key role in educating nurses by providing access to clinical data on biosimilars and to support their incorporation and appropriate use in oncology practice. Consequently, knowledge of biosimilar-related principles and policies should be incorporated into educational planning for all oncology nurse professionals [9]. A survey of 277 healthcare providers (including physicians, nurses, and pharmacists) conducted by the National Comprehensive Cancer Network (NCCN) showed that there was a suboptimal level of understanding of biosimilars and their regulation. Among the respondents, nearly half of the 71 nurses (44%) indicated that they were not at all familiar with biosimilar developments, including legislation creating the US biosimilar approval pathway. In addition, approximately one-third (31%) indicated that they would require more information before deciding on their interest level for prescribing, dispensing, or administering biosimilars in their oncology practice setting [9]. Oncologists rely strongly on their nurses and their advanced practice providers when it comes to prescribing medications and switching medications for patients. When the nurses do not feel comfortable with the biosimilar medications, they regularly encourage the oncologists not to switch the medications and to remain with the originator products.

### 4.7. Barrier to Prescribing #6: Lack of Understanding of the Rigor of the Approval Process of Biosimilars

Two of the greatest barriers to prescribers using biosimilars is lack of understanding when it comes to interchangeability and extrapolation. The FDA continues to recommend stakeholders seeking an interchangeability designation provide data on ≥3 switches between the originator and biosimilar. Data on multiple switches between a biosimilar and its innovator continue to be collected in the randomized controlled setting and remain consistent with single-switch data while not indicating a loss of efficacy or an increase in adverse events. However, limited data exist on multiple switches in the real-world setting [28]. These multiple hoops to jump through give very few biosimilars the true interchangeability designation. Interchangeability means the pharmacy is allowed to switch without the permission of the physician. Given that biosimilars will, by necessity, be manufactured in a slightly different manner from their originator product, there is concern that switching patients from a biologic to a biosimilar, or vice versa, could result in hypersensitivity reactions. Oncologists are extremely adamant that they are consulted by the pharmacy before allowing their patients to be switched to a biosimilar. For the health and safety of their patients, they are unlikely to relinquish this control any time soon. 

Extrapolation is the approval of a biosimilar for use in treatment of a medical condition used by an originator biologic, yet not directly studied in a comparative clinical trial with the biosimilar. Extrapolation of indications for biosimilars is a more complex issue because this decision and treatment may be diverse in different patients and diseases. For instance, independent contributions of complement activation and antibody-dependent cellular cytotoxicity responses are often challenging to calculate [29]. Additionally, for long-term treatments, such immune mechanisms are crucial, and the in vivo immunogenicity of these drugs should be extensively scrutinized to overcome any immune differences between the biosimilar and its originator product [29]. Extrapolations to different indications are permitted if the mechanism of action and receptors involved for various indications are the same. Due to the complexity of both interchangeability and extrapolation, many oncologists are not familiar with which biosimilars are safe and effective switches to make from the originator products.

### 4.8. Barrier to Prescribing #7: Formulary Status of the Biosimilars and Hesitancy by Providers and Institutions to Stock Multiple Drugs for the Same Indication

In the acute-care setting, biosimilars can be incorporated through the pharmacy and therapeutics (P&T) committee within the healthcare institution. This committee is primarily responsible for approving the organization’s formulary system includes pharmacists, physicians, hospital administrators, and nurses to support the medication use process. Frequently, P&T committees have been hesitant to add biosimilars because of not receiving substantial discounts from the manufacturers to make it financially lucrative. The P&T committee has also been hesitant to carry more than one medicine for the same indication because of the fear of the medicine not being prescribed by the physicians. 

The other barrier that prescribers face is that some pharmacy benefits managers (PBMs) enter into agreements where they receive substantial rebates for utilizing a specific brand/type reference product. As a result, they are often vested in dispensing the originator product as opposed to a biosimilar because they possess an inherent reduced financial incentive to offer the less expensive biosimilar [26]. Providers may not be able to adopt biosimilars if payers prefer innovator products.

### 4.9. Implications

After conducting this review, it became clear that changes need to occur in the oncology market for biosimilars to be prescribed by more physicians for more patients. It is also clear that both oncologists and their patients often do not trust the safety and efficacy of biosimilars, and they are unsure of the process that these medications go through to be approved. Biosimilar companies appear to have presumed that they would take the same route as generics and the managed care companies would force the physician’s hand by requiring step therapy and restricted formularies. However, to date, the managed care companies have not required the oncology providers to use biosimilars in the same manner as generics. This is due in large part to much fewer biosimilars entering the market and the cost savings not being as significant for the patients or for the managed care plans. Biosimilar companies have also failed to offer the resources to physicians or to the patients. Biosimilars can still penetrate the oncology market, but the biosimilar companies will need to make some adjustments to their marketing and managed care approach.

### 4.10. Recommendations

There are several recommendations that could increase the utilization of biosimilars to oncology patients. Biosimilar companies need to help physicians and patients understand the rigorous clinical trials these drugs undergo before being approved by the FDA. Physicians and patients both have concerns about switching to these medications because they do not believe the medications have undergone the clinical trials to show the safety and efficacy. A recommendation is that the biosimilars hire their own marketing team that will develop marketing plans around the value of their medications. The marketing team should also create information for the patients to review about their medications and explain the clinical trials to the physicians. If physicians can have the clinical trial papers to review, they are increasingly more probable to prescribe the medication. This would also allow the physicians to have direction on how to transition patients from originators to biosimilars and give them the confidence to explain the benefits of biosimilars to patients. This would also combat the originator companies counter detailing against the biosimilars. The biosimilar companies and their marketing team should create direct to consumer advertising. This would drive the patients into the physician offices asking about the biosimilar medication and if it is the proper medication for them. Frequently, physicians do not believe they have the time to explain the medication to the patients; however, if the patient inquiries about the medication, the oncologists are more likely to prescribe the medication. 

Another recommendation is that biosimilar companies need to be willing to negotiate with the managed care companies and pharmacy benefit managers (PBMs). These entities are searching for discounted pricing, as well as rebates, from the manufacturers. Prescription drug coverage in the United States is now dictated more by pricing than efficacy. Generics were able to penetrate the market by negotiating deals with the managed care companies and PBMs. These deals included rebates and required generic step therapy before the branded products could be used. This is a comparable approach that biosimilars want to consider ensuring their products are used instead of the originators. 

The final recommendation is the need to educate nurses and advanced practice practitioners on biosimilars. Often, this group of healthcare providers is undervalued when it comes to patients being prescribed biosimilars. Oncologists often do not believe they have the time to explain biosimilars and their efficacy to their patients. Thus, they rely on their staff to go over the types of medications to their patients. If the staff does not feel comfortable with the biosimilars, they will often ask the physician to switch back to the originator product. The nurses are key when it comes to explaining the safety and efficacy to the patients.

## 5. Conclusions

Biosimilar agents continue to play a key role in the treatment of cancer and related diagnoses. While the primary goal of clinical trials surrounding biosimilars is to demonstrate their safety and efficacy, introducing them into the standard of care continues to be dynamic process involving multiple stakeholders. This initiative could assist patients in controlling the cost of cancer care during such a traumatic treatment and recovery period. An acceptance of biosimilars by patients will depend on the level of comfort of the physicians and related efforts in educating patients during the prescribing period. Physician contentment will be contingent on additional clinical trials and related biosimilar research, thereby increasing the amount of available data surrounding their use and patient outcomes. Improving insurance coverage of new biosimilars will help increase patient access to biosimilars. Education for physicians and their staff about these identified barriers to prescribing of biosimilars will remain complex, yet imperative.

This review provided multiple areas and initiatives for future research opportunity surrounding the use of biosimilars. Future research will be needed to ensure any emerging or potential toxicological concerns are given top priority for further research and investigation to ensure optimal patient outcomes. Furthermore, investigations into the use of new biomaterials, to include new technologies such as nanoantioxidants, may also be necessary.

## Figures and Tables

**Table 1:** Summary of findings (*n* = 27).

Author(s)	Participant(s)	Barriers Leading to Prescribers and Cancer Centers Not Using Biosimilars for Patients
Leighl, B., et al., 2021 [2]	A team of US community oncologists	Institutional markup and incentives provided to providers for using the originator drug which is more expensive. Physicians not following evidence-based medicine guidelines to decide which medication should be prescribed to patients.
Nabhan, B.A., et al., 2018 [1]	Specialty pharmaceutical distribution for a hospital system	Prescribers uncertain if clinical evidence is adequate and if products are interchangeable or if indications can be extrapolated. Complex reimbursement rules for biosimilars create confusion for billing offices. Prescribers may be more willing to accept biosimilars when treating for palliative intent rather than curative intent. Patients may be reluctant to accept what they view as “generic” products.
Boccia, et al., 2017 [3]	National cancer centers in the United States, global medical affairs for Pfizer, and US government relations	Rigorous regulatory standards are needed for clinicians to consider biosimilars as acceptable alternatives to the standard of care. Long-term clinical evidence of the safety and immunogenicity of biosimilars was required for decision making about their use in chronic disorders. In some therapeutic areas, such as oncology, there is a financial incentive for both physician practices and institutions to use more expensive originator biologics rather than biosimilar drugs, as the markup and, thus, profitability to the provider are higher. The uptake of biosimilars in the USA may be hindered by uncertainties regarding the potential savings they offer and possibly by counter detailing from companies manufacturing originator biologics.
Dolan, C., et al., 2018 [4]	A variety of 376 surveyed United States oncologists	Physicians lacked technical knowledge and understanding of the effects of biosimilars. Physicians misunderstanding if biosimilars are structurally and therapeutically identical. Physician knowledge gaps regarding all aspects of biosimilars (chemical structure, difference from originator, approval process, and availability of biosimilars in the United States).
Kaida-Yip, D., et al., 2018 [5]	Department of Medicine at California Northstate University and University of Texas; Department of Surgery at Michigan State University and Texas Tech University	Concerns regarding biosimilar immunogenicity, efficacy, adverse effects when switching from a biologic to a biosimilar, and possible long-term effects. Minimal cost difference between the originator and the biosimilar.
Zack, E., 2018 [6]	Oncology nurses	Oncology nurses are not informed on their therapeutic uses, mechanisms of action, and administration considerations. Oncology nurses are not able to explain the benefits of biosimilar medications to their patients.
FDA Promotes Efficient Biosimilar Approval, 2018 [7]	Oncology peer-reviewed journal author	Lack of scientific and regulatory clarity for biosimilar development. Lack of understanding of biosimilars among clinicians, patients, and payers.
Chopra, G., et al., 2017 [8]	Clinical oncologists	Limited guidelines on extrapolation of approved indications for biosimilars. The possibility of immunogenicity events in patients during testing, and interchangeability with the originator drug. Lack of appropriate formulation and manufacturing of biosimilars. Limited awareness of the efficacy and safety of biosimilars among healthcare providers.
Mayden, H., et al., 2015 [9]	Southwest Virginia Cancer Center, Norton, Virginia; Fletcher Allen Healthcare, Burlington, Vermont; Nebraska Cancer Specialists, Omaha, Nebraska; Amgen Inc., Thousand Oaks, California	Lack of training to advanced practitioners regarding comprehensive continuing education on biosimilars to ensure public safety. Advanced practitioners need more education on how to prescribe biosimilars.
Hemmington, A., et al., 2017 [10]	Group of 327 surveyed medical specialists throughout the United States	Concerns over indication extrapolation and switching patients from an existing biologic. Lower perceived time to explain a biosimilar to a patient. Lack of efficacy data regarding the biosimilar. Lack of safety data regarding the biosimilar.
Hirsh, B.R., et al., 2011 [11]	Department of Medicine, Duke University and the Duke Cancer Institute, Durham, North Carolina.	Inadequate pharmacovigilance clinical trials. Lack of true cost savings for the managed care plan and the patients. Degree of interchangeability that will be required with biosimilars and the clinicians understanding of this clinical piece.
Abraham, J., 2013 [12]	Oncologist Cleveland Clinic	Clinicians not understanding the difference between generics and biosimilars.
Jacobs, I., et al., 2017 [13]	Proposed biosimilars were identified in 23 studies (36 publications) in oncology	For all proposed biosimilars, it should be noted that the number and quality of studies providing evidence of structural and functional similarity at the preclinical phase may not necessarily be sufficient to predict behaviors or performance in humans. Lack of evaluations of cost and potential cost savings have been performed by oncologists, institutions, and payers.
Janjigian, Y.Y., et al., 2018 [14]	Clinicians for pharmaceutical companies	Lack of understanding of the production and manufacturing process of biosimilars. Lack of knowledge regarding comparative pharmacokinetics, and efficacy and safety in a relevant therapeutic indication. Savings are not expected to be on the same level as those seen for generic drugs. Patients have concerns about receiving or switching to biosimilar treatment; they may have questions about how safe the biosimilar is, and whether it will be as effective as the originator biologic.
Kar, I., et al., 2022 [15]	Cohort of PharmDs	Changes in payor coverage. EMR challenges in preparation for future biosimilars. Lack of education for the precertification team regarding biosimilars. Lack of operational support for pharmacy inventory.
Kolbe, A.R., et al., 2021 [16]	507 surveyed healthcare clinicians from various specialties.	Gaps in prescriber knowledge and hesitancy toward biosimilars remain significant challenges for biosimilar uptake. Lack of experience prescribing biosimilars may inhibit uptake. Concerns about whether the two products would have the same expected clinical performance.
McCoy, J., et al., 2019 [17]	Comprehensive Cancer Center Physicians from Northwestern University, Chicago, IL, USA	Concerns with safety. Concerns with appropriate clinical trials for efficacy. Concerns with sufficient cost savings.
Nixon, N.A., et al., 2018 [18]	Cancer Center faculty	Concerns over how best to assess equivalence. Concerns over how to integrate biosimilars into the oncology practice. Concerns over biosimilars becoming the sole option for patients. Lack of education about biosimilars to providers.
Peters, M., et al., 2021 [19]	A team of world community oncologists	Concerns regarding accessibility and cost–effectiveness of cancer therapies. Concerns regarding quality aspects of biologics. Concerns about clinical and nonclinical trials.
Rosen, R.L., et al., 2017 [20]	Oncologists specializing in colorectal cancer	Access may be limited due to affordability in areas where out-of-pocket expense to patients is relevant. Issues with reimbursement by private insurance or the healthcare system may also impact treatment strategies. Treatment protocols or guidelines not recommending biosimilars.
Lucio, S., 2018 [21]	PharmD	Concerns with extrapolation. Concerns with interchangeability. Concerns with manufacturing and assessment methods.
Simoens, S., 2021 [22]	Department of Pharmaceuticals and Pharmacological Sciences	Healthcare professionals lack acceptance of biosimilars. Government policies and incentives.
Socinski, M.A., et al., 2015 [23]	Cohort of United States oncologists	Physicians do not consider biosimilars to be interchangeable with the reference product and, therefore, should not be automatically substituted. Misconception about extrapolation that the focus is on the clinical data alone for making the justification. Concerns about the long-term safety of biosimilars.
Tariman, J.D. 2018 [24]	Assistant professor at College of Nursing at DePaul University	Clinical safety concerns. Clinical efficacy concerns. Tolerability concerns. Interchangeability concerns. Education gaps for nurses regarding explaining biosimilars to patients.
Bhatt, V., 2018 [25]	PharmD	General unfamiliarity with key concepts of biosimilars. Education gaps for HCPs regarding variability within biosimilars, immunogenicity, and interchangeability. Limited cost savings with biosimilars.
Kvien, V., et al., 2022 [26]	Cohort of clinical professors and oncology medical directors	The reimbursement of a drug is tied to the average sales price (ASP); hence, the higher the ASP, the higher the reimbursement (Medicare Part B is set at 104.3% of ASP). In this system, a biosimilar with a lower ASP delivers a lower reimbursement than its reference product with a higher ASP. Physicians express concerns with “switching”, which is changing from the reference product or even changing back and forth between the reference product and the biosimilar agent, especially when the reason for the switch is cost-oriented. Patient reluctance to switching is usually tied to concerns over safety and efficacy.
Zinzani, P.L., et al., 2019 [27]	Cohort of clinical professors and oncology center directors	Disconnect between the biosimilars that are approved for use and those accessible in clinical practice. Complex healthcare insurance policies. Concerns over extrapolation.
